# Novel insights into the study of goblet cell hypersecretion in allergic rhinitis

**DOI:** 10.3389/fimmu.2025.1525928

**Published:** 2025-01-31

**Authors:** Xiaojia Zhu, Fengli Cheng, Hongying Duan, Sirui Fu, Changqing Zhao

**Affiliations:** ^1^ Department of Otolaryngology–Head and Neck Surgery, The Second Hospital, Shanxi Medical University, Taiyuan, China; ^2^ Shanxi Medical University, Taiyuan, China

**Keywords:** airway inflammation, allergic rhinitis, goblet cell, mucus secretion, metaplasia, neuroimmune regulation

## Abstract

Goblet cell hypersecretion is a hallmark of airway inflammation and is driven by complex neuroimmune regulation involving submucosal glands and goblet cells. Although studies have focused on mast cell degranulation as a critical driver of nasal secretion, the role of goblet cells in this process is relatively under-researched. In allergic airway inflammation, goblet cells exhibit metaplasia and hypersecretion. However, allergen exposure does not directly trigger goblet cell degranulation, raising questions regarding the underlying mechanisms of these reactions. The activation of enteric neurons promotes goblet cell degranulation by stimulating the calcitonin gene-related peptide (CGRP)–receptor active modification protein-1 (RAMP1) axis. Meanwhile, airway goblet cells express various neuropeptide receptors, and their activation by neuropeptides such as substance P and CGRP induces mucus secretion, exacerbating allergic rhinitis-associated hypersecretion. Thus, although previously less recognised, the neuron–goblet cell signalling axis plays a critical role in allergic rhinitis mucus secretion. This review highlights current research on the neuroimmune mechanisms underlying goblet cell metaplasia and degranulation, focusing on allergic rhinitis, so as to guide clinical treatment strategies.

## 1 Introduction

Goblet cells (GCs) are distributed across various organs, including the digestive tract, respiratory tract, and conjunctiva. Despite their distinct functions arising from evolutionary adaptations, GCs have common functions (1). Mucus and mucin produced by GCs and enterocytes are crucial components of the first line of defence, interacting with the immune system to maintain homeostasis (2). However, under pathological conditions, this interaction can result in various disease phenotypes. The two most prevalent diseases of the nasal mucosa are allergic rhinitis (AR) and chronic rhinosinusitis, both of which share common characteristics, including increased mucin 5AC (MUC5AC) and MUC5B secretion. Airway secretions originate primarily from GCs, glandular tissues, and vascular exudates (3). The current treatments for AR, including antihistamines, leukotriene antagonists, allergen immunotherapy, and biologics, primarily target traditional immunological pathways (4). In contrast, chronic rhinosinusitis management relies on pharmacological and surgical interventions, focusing less on the role of GCs. Therefore, a comprehensive investigation of GC function in different diseases and the composition of nasal secretions may reveal the fundamental mechanisms underlying these different hypersecretions.

Neuro-immunity encompasses the bidirectional communication pathways between the nervous and immune systems, vital for maintaining tissue homeostasis, combating infections, and modulating inflammatory responses (5). Neural sensitisation significantly increases acetylcholine release, enhancing GC secretion. Meanwhile, neuromedin U mediates eosinophil activation and increases the number of intestinal GCs, potentially impacting mucus secretion due to eosinophilic effects (6). Moreover, neuron–GC signalling via the calcitonin gene-related peptide (CGRP)–receptor active modification protein-1(RAMP1) axis protects against colitis (7).

In the airways, neural endings facilitate GC degranulation through various neuroimmune mechanisms, including those mediated by neuropeptides, neurotransmitters, and transient receptor potential (TRP) channels (8). As recently reported, current medications often prove insufficient for patients with AR exposed to physicochemical stimuli. Neuroimmune modulation of GCs likely contributes to this challenge. A deeper understanding of the neuroimmune mechanisms underlying GC metaplasia and degranulation in AR may provide valuable insights into novel therapeutic strategies for managing this condition.

## 2 GC metaplasia and degranulation-related receptors

**Figure 1:**
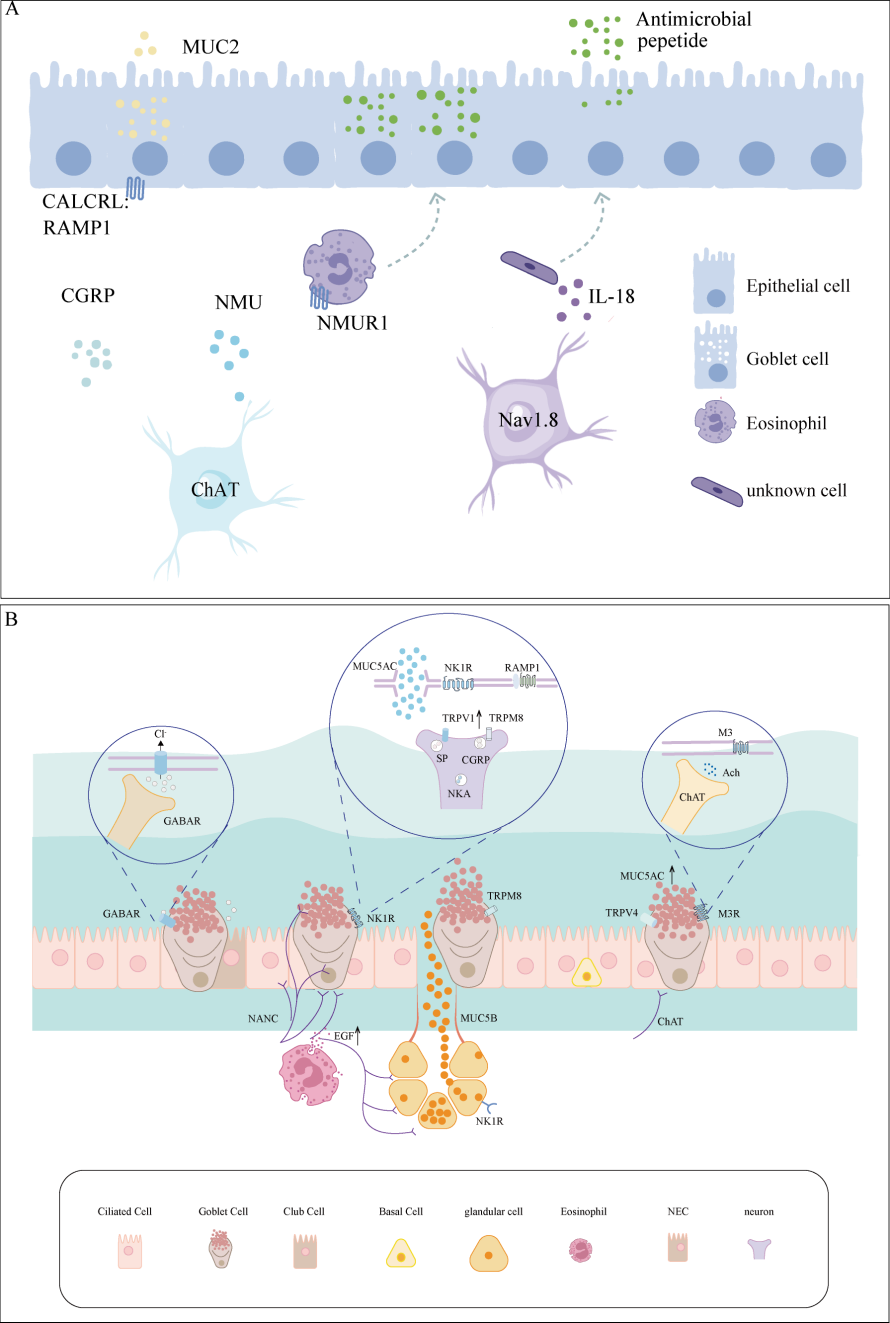
Neuro-GC interactions in the airway and intestine. **(A)** Immune crosstalk between neurons and GCs in the intestine. Intestinal neurons release CGRP and NMU, which directly or indirectly stimulate GCs, promoting MUC2 secretion. Nav1.8+ neuron-derived IL-18 orchestrates antimicrobial peptide secretion. **(B)** Immune crosstalk between neurons and GCs in the airway. The nerves, which predominantly consist of cholinergic and sensory nerve fibres, act upon GCs. External stimuli, such as temperature fluctuations and allergens, trigger the release of neurotransmitters and neuropeptides, which interact with receptors on GCs, including M3, NK1R, and GABAAR. CALCRL, calcitonin receptor-like receptor; CGRP, calcitonin gene-related peptide; ChAT, choline acetyltransferase; EGF, epidermal growth factor; GABAR, gamma-aminobutyric acid receptor; GC, goblet cell; M3, muscarinic type 3; MUC2, mucin 2; MUC5AC, mucin 5AC; NANC, non-adrenergic non-cholinergic; NKA, neurokinin A; NK1R, neurokinin-1 receptor; NMU, neuromedin U; NMUR1, neuromedin U receptor 1; RAMP1, receptor activity-modifying protein 1; SP, substance P.

The neuroimmune system is pivotal in the GC hypersecretion associated with AR. Although our understanding of the effects of GCs on AR remains limited, research on other systems provides valuable information. For example, voltage-gated sodium channel 1.8+ neurons are present near mucus-secreting GCs, where intestinal nerve–GC signalling may induce mucus secretion via the CGRP–RAMP1 axis (7). Additionally, intestinal neuronal cells release interleukin (IL)-18, which promotes mucin secretion (9). The specific mechanisms in the intestine are shown in **Figure 1A**. Mucosal barrier immunity is essential for maintaining the commensal microflora and combating infection by invasive bacteria, whereas tuft cell-derived acetylcholine regulates epithelial mucus secretion (10). Moreover, inhibiting TRP cation channel subfamily M member 4 (TRPM4) protein activity in cystic fibrosis cell lines abolished MUC5AC secretion (11). Finally, nerve–GC interactions promote allergic conjunctivitis through goblet cell-associated antigen passages (GAPs). The function of GAP as a novel therapeutic target for airway allergic inflammation warrants further investigation.

**Figure 2:**
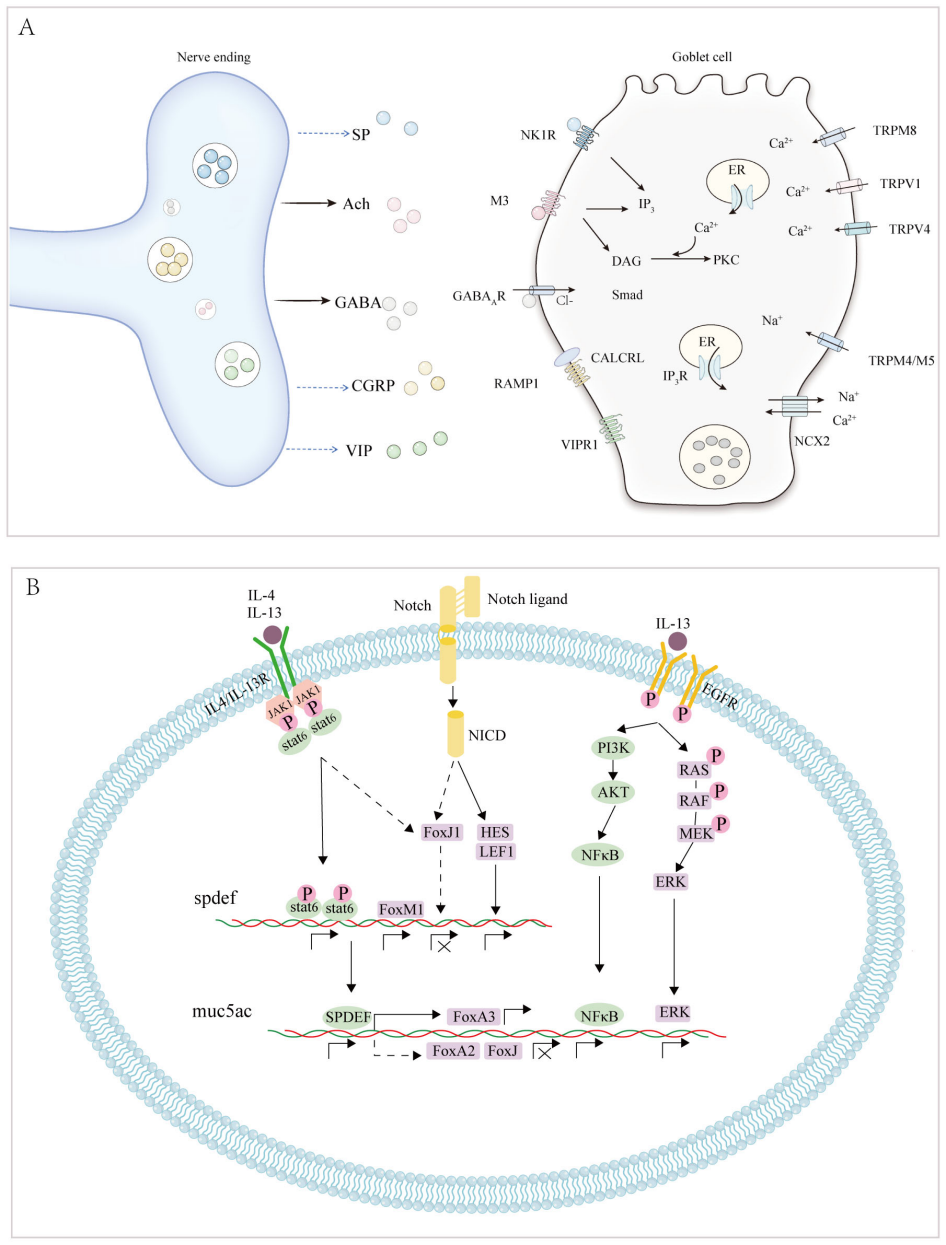
**(A)** Neuropeptides released from nerve endings and receptors on GCs are illustrated. Solid black lines highlight confirmed findings, while blue dashed lines represent findings yet to be validated. **(B)** Cellular signal transduction during GC differentiation. Muc5ac is the structural gene governing GC differentiation, and SPDEF, FOXA3, FOXA2, FOXJ1, NFκB, and ERK act as its transcription factors. The Notch signalling pathway is responsible for the differentiation of cells into secretory cells under normal conditions, while EGFR, and IL-4R are involved in the regulation of GC metaplasia in allergic rhinitis. ACh, acetylcholine; CALCRL, calcitonin receptor-like receptor; CGRP, calcitonin gene-related peptide; ER, endoplasmic reticulum; GABA, gamma-aminobutyric acid; GC, goblet cell; IP3, inositol trisphosphate; M3, muscarinic acetylcholine type 3; NCX2 (SLC8A2), solute carrier family 8 member A2; NKA, neurokinin A; NK1R, neurokinin 1R; RAMP1, receptor activity-modifying protein 1; SP, substance P; TRP, transient receptor potential vanilloid 1; VIP, vasoactive intestinal peptide. EGFR, epidermal growth factor receptor; FOXA2, forkhead box protein A2; GABA A, gamma-aminobutyric acid type A; GC, goblet cell; IL-13, interleukin 13; LEF-1, lymphoid enhancer-binding factor 1; MUC5AC, mucin 5AC; NICD, Notch intracellular domain; SPDEF, SAM-pointed domain containing ETS-like factor; STAT6, signal transducer and activator of transcription 6.

In asthma, substance P produced by airway sensory neurons amplifies allergy-induced GC hyperplasia and MUC5AC hypersecretion (12). Similarly, activating the gamma-aminobutyric acid (GABA) type A receptor, a specific chloride channel, triggers mucin release. GABA secreted by neuroendocrine cells also promotes GC hyperplasia, resulting from the increased trans-differentiation of ciliated airway epithelial cells and club cells into mucin-producing GCs following ovalbumin challenge. GCs are often located near airway sensory neuron terminals and express receptors for various neuropeptides, including muscarinic acetylcholine receptor type 3 (M3), neurokinin-1 receptor, GABA type A receptor, and vasoactive intestinal peptide (VIP) receptor 1 (13). TRP melastatin subtype 8 (TRPM8), a calcium channel, modulates intracellular calcium levels and influences GC activity, leading to MUC5AC secretion, particularly during cold exposure (14). The specific details are illustrated in **Figure 2A** and **Table 1**.

**Table 1:** Receptors on GCs in AR and other diseases.

Receptor	Type	Ligand/stimulus	Downstream pathways	Mechanism of action in AR	Roles in other organs
Cholinergic M3 receptor	GPCR	Acetylcholine	Phosphoinositide pathway and Ca^2+^ signalling	GC metaplasia and mucus secretion.	Promote mucus secretion in the intestine, airway, and conjunctiva
GABAa receptor	Ion channel receptor, specifically a chloride channel	GABA	SMAD pathway	Unknown	Asthma: GC hyperplasia
TRPM8	Ion channel, specifically a calcium channel	Physical: cold menthol and icilin	PKC, NFκB	Trigger MUC5AC secretion	Asthma/COPD: mucus hypersecretion
TRPM4/5	Sodium-selective ion channel	Ca^2+^	TRPM5 and Na^+^ channels via calcium signalling	Stimulate mucin secretion	Cystic fibrosis: MUC5AC secretion
TRPV4	Ion channel, specifically a calcium and sodium channel	Temperature, mechanical force, osmotic pressure, chemical substances	CaMK, PKC, MLCK	Detect mucus viscosity, regulate mucus production	Asthma: mucus secretion
TRPV1	Ion channel, specifically a calcium and sodium channel	Temperature, mechanical force, osmotic pressure, chemical substances	PKC	Unknown	Unknown
P2Y2	GPCR	ATP	PKC	Increase mucin secretion	Dye eye: increase mucin secretion by GCs
NK1R	GPCR	Substance P	Unknown	Increase mucus secretion GC: unknown	Asthma: SP heightens GC hyperplasia and hypersecretion of MUC5AC

AR, allergic rhinitis; ATP, adenosine triphosphate; CaMK, Ca2+/calmodulin-dependent protein kinase; COPD, chronic obstructive pulmonary disease; GABA, gamma-aminobutyric acid; GC, goblet cell; GPCR, G protein-coupled receptor; M3, muscarinic acetylcholine receptor type 3; MLCK, myosin light chain kinase; MUC5AC, mucin 5AC; NFκB, nuclear factor kappa B; NK1R, neurokinin-1 receptor; P2Y2, purinergic receptor P2Y2; PKC, protein kinase C; TRP, transient receptor potential; TRPV4, transient receptor potential cation channel subfamily V member 4.

## 3 Neuroimmune mechanisms of GC metaplasia during AR

### 3.1 Traditional immunological mechanisms of GC metaplasia

Allergens trigger GC metaplasia, and IL-13 is a critical factor driving airway allergic inflammation, GC proliferation, and metaplasia (15). IL-13 upregulates several genes involved in GC metaplasia, including SAM pointed domain-containing ETS transcription factor (*SPDEF*), forkhead box A2 (*FOXA2*), and *MUC5AC* (16–19). Specifically, it enhances SPDEF transcription, subsequently stimulating FOXA2 expression, leading to MUC5AC upregulation and stromal cell metaplasia in GCs (20).

IL-13 promotes GC metaplasia through multiple signalling pathways. For instance, circZNF652 and microRNA-141 are upregulated in patients with airway allergic inflammation, contributing to GC metaplasia by downregulating microRNA-452 and modulating IL-13 signalling (21, 22). *In vivo*, IL-13 increases *MUC5AC* transcription while suppressing FOXJ1, preventing GC apoptosis in AR (23). *In vitro*, IL-13 induces MUC5AC formation via the phosphatidylinositol 3-kinase and Janus kinases 1 (JAK1)-signal transducer and activator of transcription 6 (STAT6) pathways (24), as shown in **Figure 2B**. Thus, blocking IL-13 signalling and reducing GC metaplasia could enhance mucociliary clearance and restore the nasal epithelial structure (25).

### 3.2 Neurogenic mechanisms of GC metaplasia

Another important property of IL-13 is its ability to activate or sensitize peripheral sensory neurons. In addition to inflammatory factors’ responses, neurogenic inflammation mediated by neurotransmitters contributes to GC metaplasia regulation. The vagus nerve releases Substance P, which promotes GC metaplasia by interacting with the neurokinin-1 receptor (12). This induces GC metaplasia in the airways and promotes features such as GC hyperplasia (16). Neuroendocrine cells express GABA, and GABA type A receptors are upregulated in patients with airway allergic inflammation. GABA’s action on these receptors inhibits the SMAD pathway and promotes GC proliferation (26–28). The biological clock also helps regulate GC proliferation and differentiation (29). Animal studies (29, 30) have demonstrated more significant GC proliferation, metaplasia, and secretion in mice with disrupted circadian rhythms than those with normal rhythms. Furthermore, MUC5AC levels exhibit a circadian rhythm, indicating that it may be a therapeutic target for airway allergic inflammation. Thus, chronotherapeutics related to MUC5AC may exhibit enhanced efficacy with reduced side effects.

## 4 Neuroimmune mechanisms of GC degranulation during AR

The airways involve three neural pathways, namely sympathetic (adrenergic), parasympathetic (cholinergic), and non-adrenergic non-cholinergic (NANC) (31). GC degranulation is primarily regulated by the cholinergic and NANC sensory nervous systems (32). In the nasal mucosa, NANC nerve fibres are predominantly found in the trigeminal nerve’s C fibres, which are particularly susceptible to direct activation by allergic mediators (31). Afferent C fibres often express TRP ion channels, which promote mucin synthesis and secretion by releasing neuropeptides and neurotransmitters (12).

### 4.1 Cholinergic regulation of GC degranulation

The proximity of parasympathetic and sympathetic nerves to GCs underscores their critical role in regulating nasal secretion (33). In patients with AR, upregulation of M receptors in the nasal mucosal epithelium, vagus nerve, sensory nerve fibres, and lymphocytes facilitates acetylcholine signal transduction (34). Concurrently, a reduction in sympathetic α and β receptors leads to a neuroimmune imbalance, disrupting the typical functions of the sympathetic nervous system, which typically reduces secretions, and the parasympathetic nervous system, which promotes secretion. Parasympathetic neurons release acetylcholine, which acts directly on M3 receptors in GCs via the protein kinase C pathway, inducing MUC5AC secretion. Additionally, acetylcholine produced by tuft cells modulates epithelial fluid secretion (12, 35), highlighting the pivotal role of the cholinergic signal in mucus secretion. Moreover, clinical observations and studies have shown that a vidian neurectomy can disrupt the nerve–GC axis, demonstrating its potential for managing nasal hypersecretion in patients with AR (36).

### 4.2 NANC regulation of GC degranulation

Chemical sensation in the nasal mucosa is mediated by NANC neurons located near specialised chemosensory cells. When sensory nerve endings in the epithelium detect inhaled irritants, local or axonal motor neurotransmission through collateral ‘sensory-efferent’ pathways triggers sensory neuropeptide release. The peptides most relevant to airway mucus secretion include Substance P, neurokinin A, and CGRP. The sensory nerves and the released neurotransmitters and neuropeptides initiate GC degranulation (31). Elevated Substance P, VIP, and CGRP levels in the nasal secretions and tears of patients with AR, in conjunction with increased Substance P and VIP levels in the nasal cavity, positively correlate with visual analogue scale scores (37). Substance P, a member of the kininase family, binds to the neurokinin-1 receptor and induces airway mucus secretion (12). Animal studies have shown that Substance P released by airway sensory neurons following ovalbumin sensitisation directly promotes excessive MUC5AC secretion and GC proliferation (12). Additionally, allergens activate sensory nerve endings and amplify central nervous system signalling via CGRP, increasing the efficacy of efferent nerve terminals. This may explain why vidian neurectomies yield bilateral benefits over unilateral surgical procedures. Although GC hypertrophy was observed in an AR rat model of post-nasal neurectomy, nasal secretion was reduced due to the depletion of nerve fibres, acetyltransferase, and neuropeptides (e.g., Substance P and CGRP) in the nasal mucosa (38). Although neuropeptides are thought to promote GC secretion, the underlying mechanisms warrant further investigation.

### 4.3 Cold and heat stimuli exacerbate GC degranulation via TRP channels

Clinical manifestations indicate that temperature changes often exacerbate nasal secretions in patients with AR, highlighting the role of heat- and cold-sensitive TRP channels. Channel proteins are expressed in sensory neurons, epithelial cells, and immune cells (39).*TRPV1* mRNA expression is significantly upregulated in the nerves of a mouse model of asthma. Similarly, patients with AR had higher numbers of TRPV1-positive cells in the nasal mucosa than healthy controls. Whereas MUC5AC and mucin 5B secretion are reduced in an asthma mouse model with TRPV1 knockout (8). TRPM8 can be activated by cold stimuli or menthol, directly triggering MUC5AC secretion in epithelial cells and releasing specific amines and peptides from stromal cells (14, 40). This process facilitates mucus secretion through a paracrine mechanism, creating a positive feedback loop. Specifically, TRPM8 activation by cold stimulation induces GC degranulation through the protein kinase C pathway and Ca^2+^ influx (14, 40, 41). TRP cation channel subfamily V member 4 indirectly regulates GC degranulation by sensing mucus viscosity and controlling ciliary beating (42). ATP binds to the purinergic receptor P2Y2, activating inositol trisphosphate receptors in the endoplasmic reticulum, which increases the cytosolic Ca^2+^ concentration, leading to extracellular mucin secretion (43). ATP activation also triggers TRP cation channel subfamily M member 4/5 channels to regulate Ca^2+^ and promote Na^+^ entry, causing Na^+^/Ca^2+^ exchangers to switch modes, allowing Na^+^ efflux and additional Ca^2+^ influx to induce mucus secretion (11).

TRPV1 activation promotes cation influx across the cell membrane and sensory nerve membrane depolarisation (44). This depolarisation is amplified by voltage-gated sodium channels, which generate action potentials. *TRPV1* mRNA expression is significantly increased in the neurons of a mouse model of asthma, rendering TRPV1^+^ nerve fibres highly sensitive (45). These effects are driven by action potentials generated through TRPV1 activation and neuropeptide release (e.g., Substance P). Alterations in the expression and function of these channels can increase MUC5B secretion (46). Thus, TRPV and TRPM subfamilies represent potential therapeutic targets for controlling GC hypersecretion, particularly in sensory nerves and epithelial cells associated with airway mucus secretion. The specific details are illustrated in **Figure 1B**.

## 5 Current treatments for excessive nasal secretion

Dupilumab, a monoclonal antibody targeting IL-13Ra and IL-4Ra in clinical trials, inhibits GC metaplasia and excessive mucus secretion (47). The heat-shock protein 90 inhibitor geldanamycin reverses IL-13-induced airway GC metaplasia and improves airway remodelling (48). In human airway epithelial cells, the anticholinergic agent tiotropium attenuates IL-13-induced GC metaplasia (49). Additionally, tiotropium suppresses TRPV1 neuronal activity independently of M3 receptor blockade (49). TRPV1 agonists such as capsaicin can treat atopic rhinitis by leveraging the principle that the nasal mucosa enters a refractory period after stimulation; however, they have not significantly improved outcomes for patients with identified allergens (50). Furthermore, neuropeptides secreted by the sensory nerves and neuroendocrine cells, such as CGRP, Substance P, and GABA, can induce GC degranulation (12, 31, 51). Therefore, targeting neurotransmitter receptors in GCs is a promising therapeutic strategy. For example, stapled peptides can disrupt Ca^2+^ signalling and reduce stimulated mucin secretion (52). Although not yet explored in AR, stapled peptides represent an ‘ideal’ strategy for disrupting Ca^2+^ signalling and reducing mucin secretion.

In addition to targeting inflammatory mediators and signalling pathways, treatment methods should aim to reverse persistent GC metaplasia. This approach includes epigenetic editing to silence genes such as *SPDEF*. MicroRNAs, such as miR-141, miR-205-5p, miR-92a, and circZNF652, also target MUC5AC to alleviate mucus hypersecretion caused by allergic airway inflammation and reduce the nasal mucosal epithelial remodelling (21, 22, 53). Blocking GC metaplasia is crucial for promoting mucosal barrier restoration (23). The specific targets are listed in **Table 2**.

**Table 2:** Therapeutic targets for excessive nasal secretion.

Molecule	Target	Study design	Results	Reference
Tiotropium	Cholinergic M3 receptor	Basic research, *in vitro* and *in vivo*	1. Non-neuronal acetylcholine contributes to GC differentiation by directly affecting epithelial cells. 2. Tiotropium also fully prevented allergen-induced mucous gland hypertrophy, and partially reduced the increase in MUC5AC-positive GCs	(49)
Dupilumab	IL-13Rα and IL-4Ra	Clinical research	Inhibited GC metaplasia and excessive mucus secretion	(47)
miR-141 miR-92a circZNF652	MUC5AC	Basic research, *in vitro*	1. miR-141 regulated IL-13-induced airway mucus production 2. miR-92a contributed to blocking GC metaplasia 3. circZNF652 targeted MUC5AC to alleviate mucus hypersecretion	(21, 22, 53)
Hydrocarbon-stapled peptide	Synaptotagmin-1	Basic research, *in vitro* and *in vivo*	SP9 peptide effectively inhibited Ca^2+^-triggered mucin secretion both *in vivo* and *in vitro* by interfering with the Ca^2+^-triggered membrane fusion of synaptotagmin and SNARE proteins	(54)
Gefitinib	EGFR	Basic research, *in vitro* (NCI-H292 cells)	Gefitinib suppressed MUC5AC mRNA levels after a decrease in intracellular and secreted MUC5AC protein levels	(47)
Geldanamycin	HSP90	Basic research, *in vitro* and *in vivo*	Geldanamycin reverted GC metaplasia	(48)
IL10–MSC	Peripheral: Epithelial cells Central: Brainstem, Hippocampus, Prefrontal cortex	Basic research, *in vivo*	IL-10–MSCs significantly reduced inflammatory cell infiltration and epithelial GC numbers	(55)

EGFR, epidermal growth factor receptor; GC, goblet cell; HSP, heat-shock protein; IL, IL; M3, muscarinic acetylcholine receptor type 3; miR, microRNA; MSC, mesenchymal stem cell; MUC5AC, mucin 5AC; SNARE, soluble N-ethylmaleimide-sensitive-factor attachment protein receptor.

The neuroimmune regulatory mechanisms of GCs in other systems, such as the lower airways and intestines, have been extensively studied, including pharmacological interventions. However, the upper airways have not received adequate attention. Developing nasal sprays for delivering therapeutic agents, such as exosomes or capsaicin, or targeting genes in the nasal mucosa could be beneficial. For instance, mesenchymal stem cells overexpressing IL−10 significantly reduce the number of GCs in an allergic airway inflammation model (55). Additionally, GCs are poorly studied outside of their secretory role, and their associations with various diseases suggest the need to explore these extra functions further (56). The potential role of GCs in immune surveillance suggests that they could serve as new targets for treating allergic diseases.

## 6 Discussion

The role of nerve–GC signalling requires consideration in airway allergic diseases. Increased nasal secretion in AR is closely associated with GC metaplasia and degranulation, orchestrated by a complex network of cytokines, neurotransmitters, neuropeptides, and their respective receptors, including TRP channels. However, the precise functional mechanisms remain unclear. Zhao (57) developed and validated a chemical approach to induce high-purity GCs. Their use in functional experiments on neuropeptides and their receptors will help elucidate specific signalling pathways. In addition, introducing a hydrocarbon-stapled peptide conjugated with cell-penetrating peptides into cultured human airway epithelial cells can inhibit stimulated secretion without affecting basal secretion (54). Further breakthroughs in AR could provide multidimensional approaches for treating mucus hypersecretion diseases, including asthma, chronic obstructive pulmonary disease, and cystic fibrosis.

Although the functions of GCs in the gut, conjunctiva, and airways are distinct under pathological conditions, they all imprint the central nervous system, which regulates peripheral diseases (58). For instance, similar to the storage of immune memory in the insular cortex following dextran sodium sulphate-induced colitis, DBH^+^ neurons in the solitary tract nucleus modulate airway hyperresponsiveness in asthma (59, 60). However, phenotypic changes in peripheral neurons during airway inflammation and their impact on GC function remain largely unknown. Thus, we introduced the nose–brain axis (61), building on pioneering research in related fields. Adaptations of the central nervous system in AR and their effects on GC metaplasia and degranulation are not fully understood. Shifting the research focus from peripheral to central mechanisms offers a transformative perspective on AR pathogenesis.

GCs are not merely mucus barriers but also immune barriers, acting as the first line of defence against airway mucosal immunity. Previous research has been limited to the immunological aspects; however, future basic and clinical studies should focus on the neuroimmunological regulation of GCs. Current treatments for airway hypersecretion primarily target immune factors such as IL-13. More in-depth research on GCs is urgently needed, and new treatment strategies specifically targeting GCs are essential.
